# The Interplay of Raloxifene and Sonochemical Bio-Oss in Early Maxillary Sinus Bone Regeneration: A Histological and Immunohistochemical Analysis in Rabbits

**DOI:** 10.3390/medicina59091521

**Published:** 2023-08-23

**Authors:** Anderson Maikon de Souza Santos, Rodrigo dos Santos Pereira, Pietro Montemezzi, Rafael Coutinho Mello-Machado, Roberta Okamoto, Roberto Sacco, Paulo Noronha Lisboa-Filho, Michel Reis Messora, Carlos Fernando Mourão, Eduardo Hochuli-Vieira

**Affiliations:** 1 Department of Diagnostic and Surgery, Araçatuba School of Dentistry, Sao Paulo State University, Sao Paulo 16066-840, Brazil; 2 Department of Oral & Maxillofacial Surgery, University of Grande Rio—UNIGRANRIO, Rio de Janeiro 25071-202, Brazil; 3 Department of Dentistry, San Raffaele Hospital, 20132 Milan, Italy; 4 Department of Implant Dentistry, Universidade Iguaçu, Nova Iguaçu, Rio de Janeiro 26260-045, Brazil; 5 Department of Basic Sciences, Araçatuba School of Dentistry, Sao Paulo State University, Sao Paulo 16066-805, Brazil; 6 Department of Oral Surgery, Division of Dentistry, School of Medical Science, The University of Manchester, Manchester M13 9PL, UK; 7 Department of Physics, School of Sciences, Sao Paulo State University, Bauru 17033-360, Brazil; 8 Department of Oral and Maxillofacial Surgery and Periodontology, School of Dentistry of Ribeirao Preto, University of Sao Paulo, Ribeirao Preto 14040-904, Brazil; 9 Department of Periodontology, Tufts University School of Dental Medicine, Boston, MA 02111, USA; 10 Department of Diagnostic and Surgery, Araraquara School of Dentistry, Sao Paulo State University, Sao Paulo 14801-385, Brazil

**Keywords:** bone graft, bone regeneration, Bio-Oss, raloxifene

## Abstract

The study aimed to assess the efficacy of using Raloxifene with ultrasonic processing to enhance Bio-Oss^®^, a bone graft substitute, for maxillary sinus bone height reconstruction. A total of 24 rabbit maxillary sinuses were distributed into three groups, each receiving different treatments: Bio-Oss^®^ only, sonicated Bio-Oss, and sonicated Bio-Oss^®^ with Raloxifene. Surgical procedures and subsequent histomorphometric and immunohistochemistry analyses were conducted to evaluate the bone formation, connective tissue, and remaining biomaterial, as well as the osteoblastic differentiation and maturation of collagen fibers. Results indicated that the sonicated Bio-Oss^®^ and Bio-Oss^®^ groups showed similar histological behavior and bone formation, but the Raloxifene group displayed inflammatory infiltrate, low bone formation, and disorganized connective tissue. The statistical analysis confirmed significant differences between the groups in terms of bone formation, connective tissue, and remaining biomaterial. In conclusion, the study found that while sonicated Bio-Oss^®^ performed comparably to Bio-Oss^®^ alone, the addition of Raloxifene led to an unexpected delay in bone repair. The findings stress the importance of histological evaluation for accurate bone repair assessment and the necessity for further investigation into the local application of Raloxifene. Future research may focus on optimizing bone substitutes with growth factors to improve bone repair.

## 1. Introduction

The process of bone regeneration is a truly remarkable and awe-inspiring natural phenomenon that allows the body to effectively repair and replace lost or damaged bone tissue. Its importance cannot be overstated, as it is critical not only for healing fractures and injuries but also for addressing a range of clinical scenarios [1,2,3]. In the world of dentistry, particularly in the area of dental implants, the ability to regenerate bone is absolutely essential [1,2]. The maxillary posterior area of the mouth presents a number of significant challenges, including natural anatomical changes such as maxillary sinus pneumatization. The implementation of dental implants as a strategy for rehabilitation in the maxillary posterior region frequently encounters limitations due to the pneumatization of the maxillary sinus [1,2,3].

This anatomical alteration considerably diminishes the height of bone available for a successful implant procedure. Historically, an array of techniques and various bone substitutes have been meticulously investigated to address and mitigate this challenge of bone height restoration [1,2,3]. To ensure successful dental implant procedures, researchers must prioritize the development and utilization of innovative techniques and materials that effectively enhance bone regeneration.

Among the array of potential substitutes, autogenous bone grafts have been identified as the most suitable due to their unique triad of osteoinductive, osteoconductive, and osteogenic properties [4]. Nonetheless, the associated comorbidities resulting from the requirement of a second surgical procedure to harvest this bone graft have curtailed its widespread adoption [4,5]. An alternative bone substitute, Bio-Oss^®^, has demonstrated promising outcomes across diverse study models; however, it solely possesses osteoconductive properties [6,7]. In addition, Bio-Oss^®^ is one of the most commonly used bone substitutes in bone grafting research [4,5,6,7,8,9].

In the search for bone substitutes that present not just osteoconductive properties but also osteoinductive qualities, several research studies have suggested the incorporation of proteins integral to bone metabolism and repair. This includes recombinant human Bone Morphogenetic Protein-2 (rhBMP-2), a protein that encourages bone formation, and recombinant human Vascular Endothelial Growth Factor (rhVEGF), which promotes angiogenesis, both crucial for bone healing [4,8,9]. A novel proposition that has emerged in recent years is the association of bone substitutes with medicines such as Raloxifene, offering a potential path to enhance the efficacy of bone substitutes [10].

Raloxifene, a non-steroidal derivative of benzothiophene, acts as a selective modulator of estrogen receptors. Its therapeutic use in the management of osteoporosis has been shown to concurrently reduce the risk of breast cancer [11,12]. Additionally, Raloxifene has exhibited a significant influence on osteogenesis, including stimulation of pre-osteoblastic cells, promotion of extracellular matrix mineralization, modulation of the WNT/B-catenin pathway, a crucial intracellular signaling cascade in bone metabolism, and RUNX 2 (Runt-related transcription factor 2), a key transcription factor in osteoblast differentiation, enhancement of osteocalcin expression, increase in overall bone volume, and reduction in bone porosity [13].

Furthermore, several investigations have observed a positive effect of Raloxifene on peri-implant bone repair in osteoporotic rats, as well as in the optimization of alveolar bone healing [14,15,16,17]. The systemic administration of Raloxifene, however, has been reported to increase the risk of thromboembolism events, as indicated by Adomaityte et al. [18] and the American College of Osteoporosis [19]. Yet, the scientific literature presently lacks studies examining the systemic implications of the local administration of this medication.

In a noteworthy study by Gomes-Filho et al. [20], Raloxifene was successfully integrated with bioactive glass through an ultrasonic technique, demonstrating promising results for the treatment of rat calvaria critical defects. Nevertheless, limited research has delved into its potential for addressing more substantial bone defects, such as those found in maxillary sinus bone reconstruction. Given the novelty of combining Raloxifene with bone substitutes, it is imperative to assess its performance when paired with widely used bone grafts in extensive bone defects.

The central objective of this study, therefore, is to evaluate the potential of Raloxifene, used in conjunction with ultrasonic processing, to functionalize Bio-Oss^®^, thereby enhancing the efficacy of bone grafts for the reconstruction of maxillary sinus bone height in the initial bone healing process.

The study is guided by a hypothesis, which is:

**H_0_.**  *(null) The addition of Raloxifene will not contribute to the enhancement of new bone formation in rabbits’ maxillary sinus lifting procedures.*

## 2. Materials and Methods

The present study delineated herein was conducted meticulously, fully adhering to the Animal Research: Reporting of In Vivo Experiments (ARRIVE) guidelines [21], which provide a robust framework for ensuring transparency and reproducibility in animal research, with a staunch commitment to enhancing the quality of the work undertaken. This adherence was not merely for compliance but as an integral part of the study design, with a profound respect for ethical considerations and scientific integrity.

The study was subjected to a comprehensive review by a respected ethics committee —the ethics committee of Araçatuba Dental School, a prestigious institution under the banner of Universidade Estadual Paulista (UNESP). This committee, which is known for its stringent ethical standards and rigorous assessment protocols, granted its official approval for this study, thereby ensuring that the investigation complied with all pertinent ethical guidelines. This approval is signified by the unique identification number 350-76-2019, under which the study was registered.

### 2.1. Number of Samples

A power analysis was performed using data from Caneva et al. [22] to determine the number of maxillary sinuses required in each group. A minimum of 7 rabbit maxillary sinuses was determined based on a mean difference of 17.9, a standard deviation of 10.1, a one-tailed test, a significance level of 5%, and a power of 80%.

### 2.2. Biomaterial Preparation

The method used to prepare and analyze the biomaterial (Bio-Oss^®^ Sonicated) followed the protocol established by Lisboa-Filho et al. [10]. Raloxifene was added to 20% of the total volume during the sonication of Bio-Oss^®^. For every gram of prepared material, 0.8 g of Bio-Oss^®^ and 0.2 g of Raloxifene in its solid form were used. Ultrasonic processing was applied to the two components using a Sonics^®^ VCX-750 device (Sonics & Materials, Inc., Newtown, CT, USA) for around 3 min with 750 W power, 20 kHz frequency, and 40% of the nominal amplitude of the equipment (450 W/cm^2^). Milli-Q^®^ (Millipore, Burlington, MA, USA) ultrapure water was added to obtain a uniform system and to reduce particle size. The samples were then dried at 60 °C for 8 h and sterilized using ultraviolet radiation. Bio-Oss^®^ sonication was also performed without Raloxifene. After the ultrasonic procedure, the grafts were stored in a sterile container for sinus bone augmentation in rabbits.

### 2.3. Surgical Procedures

Twelve 6-month-old rabbits (Oryctolagus cuniculus) weighing between 3 and 4 kg were used in the study and were distributed into three groups as follows:

Group 1: 8 maxillary sinuses grafted with Bio-Oss^®^ (BO)
Group 2: 8 maxillary sinuses grafted with sonicated Bio-Oss^®^ (BS)
Group 3: 8 maxillary sinuses grafted with sonicated Bio-Oss^®^ with Raloxifene (4:1) (BR)

To carry out the surgery, the animals were fasted for 8 h and then given an anesthetic injection of xylazine (Dopaser^®^, Laboratory of Brazil Ltd., São Paulo, Brazil) at 6 mg/kg and ketamine (Vetaset^®^, Fort Dodge, Saúde Animal LTDA, Campinas, Brazil) at 10 mg/kg. To supplement the anesthesia, 0.3 mL/kg of 2% Mepivacaine Hydrochloride solution with epinephrine 1:100,000 (Mepiadre 100^®^, DFL LTDA, Rio de Janeiro, Brazil) was injected along the midline of the nasal dorsum. The surgical site was prepared by shaving the nasal dorsum of the animals and sterilizing it with 10% degerming polyvinyl pyrrolidone iodine. In order to access the sinus cavity, a linear incision of 50 mm was made in the midline of the animal’s nasal dorsum [23]. The skin and periosteum were carefully detached to expose the nasal bone and internasal suture. A circular window with a diameter of 5 mm was then created bilaterally to the midline of the nasal dorsum, approximately 20 mm before the nasofrontal suture and 10 mm laterally to the midline, using a 5 mm diameter trephine drill to mark the area and a spherical diamond drill No. 1011 (KG Sorensen^®^, Cotia, São Paulo, Brazil) to complete the osteotomies. These tools were mounted on a 20:1 (Kavo^®^ do Brasil, Joinvile, Brazil) and connected to an electric motor that was controlled for rotation (model BLM 600 plus, Driller^®^, Jaguaré, São Paulo, Brazil) at a speed of 1500 rpm. The procedure was performed with a sterile 0.9% saline solution (Darrow, Rio de Janeiro, Brazil) for irrigation. Once the window was made in the maxillary sinus, the sinus membrane was detached and elevated using specialized curettes (Neodent^®^, Curitiba, Paraná, Brazil). The grafts were prepared and packed into the surgical sites in a randomized and blinded manner, according to their distribution in the groups. The muscles and periosteum were repositioned using 5-0 Polyglactin 910 thread (Vicryl 5-0^®^, Ethicon, Johnson & Johnson, São José dos Campos, Brazil) through simple interrupted sutures. For the skin, 5-0 nylon thread (Ethilon 5-0, Ethicon Johnson & Johnson) was used. After the procedure, the animals received an intramuscular dose of 20,000 IU of penicillin G benzathine (Pentabiotic, Veterinário Pequeno Porte, Fort Dodge Animal Health Ltd., Campinas, Brazil) and 1 mg/kg/day of dipyrone sodium. The animals were euthanized by anesthetic overdose after a period of 14 days.

### 2.4. Histomorphometric Analysis

The samples were fixed in a 10% formaldehyde solution for 48 h and washed in running water for 24 h. First, the specimens were decalcified in an EDTA solution for 8 weeks. Then, they were embedded in paraffin and sliced coronally into 5 μm thick sections to observe both maxillary sinuses per slide. Finally, the sections were stained with hematoxylin and eosin. The histomorphometric analysis of the bone formation, connective tissue, and remaining biomaterial was performed using the Merz grid [24] under a light microscope with a digital camera (JVCTK 1270 Color Video Camera) at a magnification of ×12.5.

### 2.5. Immunohistochemistry Analysis

To assess osteoblastic differentiation, we conducted an immunohistochemical test by utilizing a primary polyclonal goat antibody against Runx 2 (Goat anti-Runx2—Santa Cruz Biotechnology, SC8566, Santa Cruz, CA, USA) [25]. The team employed the immunoperoxidase detection method and amplified the signal from the reaction through incubation in avidin and biotin (ABC standard kit, Vector Laboratories, Burlingame, CA, USA). For the reaction revelation, we used Diaminobenzidine (Dako Laboratories, Santa Clara, CA, USA) and performed counterstaining with Harris hematoxylin. After dehydration through xylene, the researchers mounted coverslips over the sections to evaluate and utilize a scoring system in accordance with dos Santos Pereira et al. [26], which was performed by a single evaluator who had been previously calibrated. Scores were in the range of 0 (absence of staining), 1 (low staining), 2 (moderate staining), and 3 (intense staining).

### 2.6. Collagen Fibers Maturation

Picrosirius red staining was performed to analyze collagen fiber maturation, and the areas of each type of fiber (green and red) were measured using a polarized light microscope (Leica QWin V3, Leica Microsystems, Wetzlar, Germany) and its software [27]. Green fibers were considered immature, while red fibers were considered mature collagen fibers (type 1).

### 2.7. Statistical Analysis

The normality of the data was assessed using the Shapiro–Wilk test. Parametric data were analyzed using ANOVA comparison tests followed by Tukey’s multiple comparison tests, and non-parametric data were analyzed using the Kruskal–Wallis test (GraphPad Prism 8, San Diego, CA, USA). A *p*-value < 0.05 was considered statistically significant.

## 3. Results

### 3.1. Histomorphometric Analysis Results

**Figure 1:**
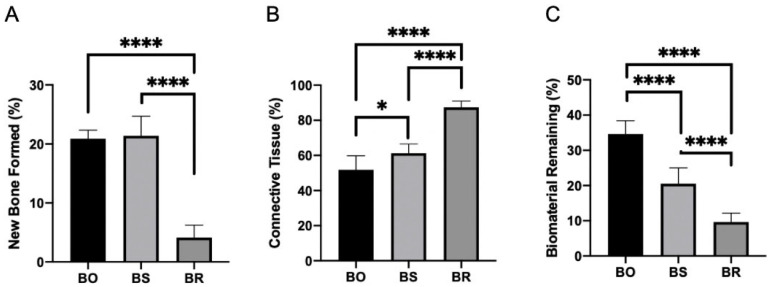
Comparative evaluation of the efficacy of three distinct biomaterials (BO, BS, and BR) in the process of bone formation. The chart provides a quantified representation of the bone formation (**A**), connective tissue (**B**), and biomaterial remaining (**C**) percentages for each group. Considering the significance * *p* < 0.05 and **** *p* < 0.0001.

**Figure 2:**
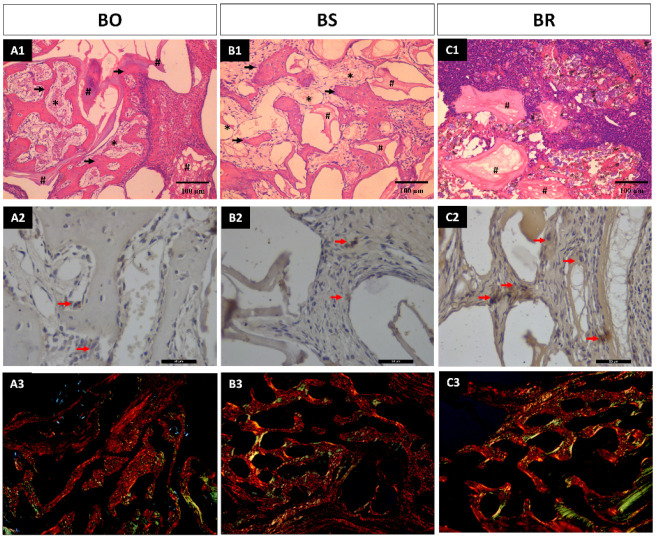
Slides of the biological responses across three biomaterial groups: BO, BS, and BR. (**A1**,**B1**,**C1**) lines show the histological evaluation (arrows mean new bone formed; * means connective tissue; and # means biomaterial remaining). (**A2**,**B2**,**C2**) lines show the results from immunohistochemistry demonstrating varying levels of Runx 2 staining in the groups evaluated (red arrows indicating positive immunostaining for Runx 2 cells; and (**A3**,**B3**,**C3**) line showing the collagen fiber maturation analysis).

The study aimed to compare the effectiveness of three different biomaterials (BO, BS, and BR) in bone regeneration. Results showed that BO and BS groups had similar behavior in histology and bone formation, with 20.87% ± 1.34 and 21.37% ± 3.33 of bone formed, respectively. However, the connective tissue presented different percentages, with 51.75% ± 8.69 in BO and 61.25% ± 5.23 in BS, while biomaterial remaining was 34.62% ± 3.54 and 20.50% ± 4.50, respectively (Figure 1). Contrarily, the BR group exhibited signs of inflammatory infiltration and lower rates of bone and vessel formation, alongside disorganized connective tissue. This led to a mere 4.12% ± 2.10 of bone formation, coupled with an abundant 87.37% ± 3.54 of connective tissue, and a residual 9.62% ± 2.55 of the remaining biomaterial. Statistical analysis indicated significant differences in bone formation (*p* < 0.0001), connective tissue (*p* < 0.0001), and biomaterial remaining (*p* < 0.0001) between the groups (Figure 2). Specifically, Tukey’s multiple comparison tests revealed significant differences between BO and BR (*p* < 0.0001) and BS and BR (*p* < 0.0001) for bone formation; BO × BS (*p* = 0.011), BO × BR (*p* < 0.0001), and BR × BS (*p* < 0.0001) for connective tissue; and all groups (*p* < 0.0001) for biomaterial remaining (Figure 2A1,B1,C1). Thus, the null hypothesis (H_0_) was accepted.

### 3.2. Immunohistochemistry Analysis

Positive immunostaining was observed in all groups. However, groups BO and BS exhibited low staining (“1”) with only a few positive cells for Runx 2 in close proximity to the bone formation. On the other hand, group BR showed moderate staining (“2”) with positive cells in the connective tissue and adjacent to areas of initial bone formation. These findings are consistent with the histological results, where the group BS had a low rate of bone formation (Figure 2A2,B2,C2).

### 3.3. Collagen Fibers Maturation Results

The mean for mature collagen fibers was 14.83 ± 8.61 pixels for group BO; 19.95 ± 8.06 for the group BS; and 26.85 ± 13.59 for group BR with no statistical significance (*p* = 0.307). The median for the immature collagen fibers formation was 0.84, 2.29, and 4.21 for groups BO, BS, and BR, respectively, also with no statistical significance (*p* = 0.211). Similar image results were found for the three groups in both analyses (Table 1) (Figure 2A3,B3,C3).

## 4. Discussion

This research initiative aimed to explore innovative methods for enhancing outcomes in maxillary sinus bone height reconstruction procedures to expand the existing body of knowledge concerning bone graft optimization. The main topic of the study was an in-depth examination of the potential of augmenting Bio-Oss^®^, a widely recognized biomaterial in bone repair, using ultrasonic processing. Furthermore, the study delved into the implications of combining this biomaterial with Raloxifene, evaluating its efficiency in bone regeneration during rabbit maxillary sinus augmentation. Remarkably, in the pivotal early bone healing timeframe of 14 days, the results revealed unexpected patterns. Contrary to the initial hypothesis that suggested Raloxifene’s integration might bolster the bone healing trajectory, it was discerned that its combination with ultrasonic processing could inadvertently reduce the speed of the healing process. This revelation underscores the multifaceted nature of bone regeneration and the imperative for meticulous evaluation of new techniques and combinations.

While the bone repair process is known to be complex and multifaceted, involving numerous biological and chemical factors [8,9,10,11,12], it was hypothesized that the addition of Raloxifene would synergize with ultrasonic processing to promote bone repair. However, the evidence garnered from this study seems to indicate otherwise. There could be several explanations for this result, ranging from the inherent prothrombotic properties of Raloxifene to potential interactions with other substances present during the healing process [14,15,16]. Therefore, further studies would be instrumental in shedding light on this unexpected delay in bone repair when Raloxifene is incorporated into the treatment protocol.

The results pointed out that the BR group, which underwent Raloxifene treatment, did not produce the desired optimal results. On the other hand, the BS group, which was not exposed to Raloxifene, surprisingly yielded promising and robust outcomes. Both the Bio-Oss^®^ only (BO) and Bio-Oss^®^ with ultrasonic treatment (BS) groups demonstrated successful bone repair, a finding that aligns well with previous preclinical [6,22] as well as clinical [28] investigations endorsing the osteoconductive properties of Bio-Oss^®^.

Of particular note, the BR group showed a significant inflammatory infiltration during the examination period, along with disorganized connective tissue. This observation suggested a delay in the bone repair process within this group. The outcome might be linked to the prothrombotic properties typically associated with Raloxifene. This drug is known to enhance clotting factors while simultaneously lowering the concentration of antithrombin and protein C [18]. As a result, subsequent research is essential to deepen the understanding of the mechanism of action of Raloxifene when applied locally.

The sole study so far that explored the integration of Raloxifene with a biomaterial via ultrasonic processing, conducted by Lisboa-Filho et al. [10], reported successful bone repair of critical defects in rat calvaria using an 80% biomaterial to 20% Raloxifene ratio. Yet, no statistically significant difference was established. This study relied solely on microtomographic analysis, a technique with inherent limitations. An accurate evaluation of bone repair necessitates a histological examination, as demonstrated in this study.

Raloxifene has previously been reported as a supplement to biomaterials in the repair of critical defects in rat calvaria [20]. Its combination with autogenous bone in a 50% ratio has also been tested in both preclinical and clinical studies, but the benefits over the standalone biomaterial were not significantly evident [29,30,31,32]. Concurrent research is exploring the possibility of integrating bone substitutes with growth factors to optimize bone repair [8,9,33]. A study by Mu et al. (2020) [33] investigated the combination of xenografts with Injectable Platelet-rich Fibrin, discovering this combination significantly improved bone repair parameters both in vitro and in vivo, particularly in the early stages of sinus membrane elevation surgery in rabbits.

When evaluating biomaterials for therapeutic purposes such as bone repair, we cannot only focus on the material’s intrinsic properties or biochemical effects. It is vital to account for wider biocompatibility aspects that dictate outcomes after implantation. A crucial factor here is the foreign body response [34]. For example, the BR group in the present study showed notable inflammatory infiltration, indicative of the body’s reaction to an unfamiliar substance. Such a response, especially if exacerbated by the combination of biomaterials with drugs like Raloxifene, can impede the bone healing process. This interference is apparent from the disorganized connective tissue observed, suggesting a negative impact from the foreign body response.

In contrast, building on previous research [35], it is interesting to observe that the effectiveness of Raloxifene in promoting bone growth might vary depending on the context. A prior study by our team, using a different xenograft, found a 20% improvement in bone formation with Raloxifene, especially over an extended 42-day healing period. This contrasts with our current findings, even though the same rabbit sinus model was employed. This raises an important question: does the specific bone graft used have the potential to influence the bone formation process? It seems possible that different xenografts might alter Raloxifene’s therapeutic effect, amplifying the foreign body response and affecting bone regeneration. The increased inflammatory response seen in the BR group supports this theory. For a deeper understanding and to fully realize Raloxifene’s potential in bone regeneration, future studies should delve into its interaction with various xenografts and the ensuing foreign body reactions.

Despite this study providing insights into the initial bone healing period during maxillary sinus lifting and the immunohistochemistry of a specific cell type, it is worth acknowledging certain limitations. Experimental studies of this nature often require multiple evaluations, especially when biomaterials are subjected to physical and chemical changes. Therefore, additional immunostaining for TRAP, Osteocalcin, and VEGF is essential, coupled with long-term assessments to evaluate the effects of Raloxifene on bone healing.

The findings suggested that in the context of rabbit maxillary sinus lifting, the combination of Raloxifene and Bio-Oss^®^ through ultrasonic processing did not outperform the use of Bio-Oss^®^ alone for bone repair. This study underscores the significance of histological evaluation for an accurate assessment of bone repair and signals the need for further research into the local mechanism of action of Raloxifene. Ongoing studies are exploring the integration of bone substitutes with growth factors to achieve optimal biomaterial results for enhancing bone repair.

## 5. Conclusions

Within the 14-day early bone healing period, the study unveiled some surprisingly unexpected results. The employment of Raloxifene alongside ultrasonic processing unpredictably delayed bone repair. On the contrary, the group subjected solely to ultrasonic processing, without Raloxifene, presented favorable tissue organization conducive to bone formation. These findings suggest that Raloxifene should be further studied for its impact and highlight the potential of ultrasonic processing as an independent method for promoting early-stage bone healing.

## Figures and Tables

**Table 1:** Identification of the results of histomorphometric analysis and collagen fibers maturation.

	BO	BS	BR
Bone Formed (%)	20.87 ± 1.34 ^A^	21.37 ± 3.33 ^AB^	4.12 ± 2.10 ^C^
Connective tissue (%)	51.75 ± 8.69 ^A^	61.25 ± 5.23 ^B^	87.37 ± 3.54 ^C^
Biomaterial Remining (%)	34.62 ± 3.54 ^A^	20.50 ± 4.50 ^B^	9.62 ± 2.55 ^C^
Mature Collagen Fibers (pixels)	14.83 ± 8.61 ^A^	19.95 ± 8.06 ^A^	26.85 ± 13.59 ^A^
Immature Collagen Fibers (pixels)	0.84 ^A^	2.29 ^A^	4.21 ^A^

The superscript letters A, B, and C following the data in the table denote the statistical significance of the differences between the groups BO, BS, and BR for each parameter. In addition, similar letters have no statistical difference.

## Data Availability

No new data were created or analyzed in this study. Data sharing is not applicable to this article.
